# The Effects of Different Levels of Dietary Protein and L-Carnitine on Blood Sugar and Lipids of the New GIFT Strain of Juvenile Nile Tilapia (Oreochromis niloticus)

**DOI:** 10.1100/tsw.2009.129

**Published:** 2009-11-01

**Authors:** Gang Chen, Minghui Zhang, Jiandong Zhang, Hongbiao Dong, Hui Zhou, Baogui Tang, Jiansheng Huang, Gang Shi, Ling Jiang, Zhaohe Wu

**Affiliations:** College of Fisheries, Guangdong Ocean University, Zhanjiang 524025, China; Guangdong South China Sea Key Laboratory of Aquaculture for Aquatic Economic Animals, Zhanjiang 524025, China

**Keywords:** blood sugar, dietary protein, L-carnitine, lipid, new GIFT strain Nile tilapia

## Abstract

The new GIFT (Genetically Improved Farmed Tilapia) strain of Nile tilapia is a popular cultivated fish in Asia, but intensive aquaculture using nutritionally imbalanced feed has led to disorder of lipid metabolisms. An 8-week feeding experiment was conducted in order to assess the effects of different levels of L-carnitine (0, 200, 400, 600, and 800 mg/kg) and dietary protein (22, 25, and 28%) on blood sugar and blood lipid contents of the new juvenile GIFT strain of Nile tilapia. Results showed that dietary protein and L-carnitine had significant influences on glucose (GLU), high-density lipoprotein–cholesterol (HDL-C), total cholesterol (TC), triglyceride (TG), and low-density lipoprotein–cholesterol (LDL-C) in the blood serum. The contents of GLU and HDL-C increased with the increases in dietary protein and L-carnitine levels, while the contents of TC, LDL-C, and TG decreased with the increases in dietary protein and L-carnitine levels. The interactive effect of both dietary protein and L-carnitine was most significant on GLU (*p* = 0.0001), followed by TG (*p* = 0.001), TC (*p* = 0.005), HDL-C (*p* = 0.056), and LDL-C (*p* = 0.109). These results suggested that high levels of dietary protein and L-carnitine supplementation reduce blood lipids and the burden of the fish liver.

## INTRODUCTION

L-carnitine (l-h-hydroxy-g-N,N,N-trimethylaminobutyric acid) is a nonessential organic nutrient that is required for the entry of long-chain fatty acids into mitochondria for β oxidation. L-carnitine serves as a growth promoter, helping in the utilization of high fat levels in the diet and thus providing a protein-sparing effect. Numerous studies have been conducted to assess the roles of L-carnitine on enhancing animal production and improving body composition for human consumption[1,2]. L-carnitine supplementation in animal diets also influences blood sugar and lipid levels[3,4,5,6]. However, the effects of L-carnitine supplementation and protein level on different blood sugar (glucose, GLU and triglyceride, TG) and cholesterol lipids (high-density lipoprotein–cholesterol, HDL-C, total cholesterol, TC, and low-density lipoprotein–cholesterol, LDL-C) have rarely been studied on cultivated fish[1,2].

The new GIFT (Genetically Improved Farmed Tilapia) strain of Nile tilapia (*Oreochromis niloticus*) is the most widely cultivated tilapia in Asia[7]. Intensive aquaculture of tilapia using nutritionally imbalanced artificial diets has been shown to lead to the disorder of lipid and blood sugar metabolism[8]. L-carnitine may help lipid metabolism and reduce fatty liver diseases. The objectives of this study were to assess simple and interactive effects of dietary protein and L-carnitine levels on blood sugar and lipids of the new GIFT strain of juvenile Nile tilapia, using a two-factorial experiment design. The changes in GLU, HDL-C, TC, TG, and LDL-C in blood serum were measured in order to determine the level of effects of both protein and L-carnitine, and to provide a physiological basis for the reduction of body and liver lipids.

## MATERIALS AND METHODS

### Experimental Fish

Juveniles of the new GIFT strain of Nile tilapia were obtained from Wuchuan County Tilapia Nursery, Guangdong, China on June 2007. Healthy sexually reversed males (body length = 1 cm) were cultivated in stainless-steel glass tanks (0.75 $m^3$). Prior to the experiment, all fish were starved for 48 h, weighed (average initial weight: 4.04 ± 0.41 g; average body length: 6.17 ± 0.20 cm), and then randomly placed into stainless-steel glass tanks (0.25 $m^3$) with a density of 15 fish per tank. Each treatment consisted of triplicate tanks.

### Experimental Feeds

The feeds used in this study were formulated in our laboratory. There were three levels of protein of 22, 25, and 28% labeled as $CP_1$, $CP_2$, and $CP_3$, and five levels of L-carnitine (0, 200, 400, 600, and 800 mg/kg) at each protein level with labels as $CP_{1-1}$, $CP_{1-2}$…, $CP_{2-1}$, $CP_{2-2}$…, $CP_{3-1}$…$CP_{3-5}$. The treatment group with 0 mg/kg of L-carnitine supplementation served as control. Feeds with 0 mg/kg of L-carnitine served as controls.

The feed was composed of fish meal, soybean, rapeseed, and cottonseed meals as sources of protein, and corn, wheat bran, and wheat middling as sources of carbohydrate. Fish oil and soybean oil were added to provide basic fatty acids, dicalcium phosphate as the source of phosphate, composite vitamin and minerals (Table 1). All raw materials were powdered, mixed to homogeneity using a model B30 multipurpose blender, and then further mixed with fish oil and water. The feed was pelleted at diameters of 1.0 and 2.0 mm, respectively, using a model F-26 (III) pellet mill. The pellets were air dried and stored in a refrigerator at 4°C before use. Feed ingredients were analyzed for proximate composition by standard methods[9].

### Experimental Management

The experiment began on July 15, 2007 and was terminated on September 8, 2007. During the experiment period, feed was given to the experimental fish twice a day (9:00 a.m. and 3:00 p.m., local time) with a total amount of 3–5% of the fish weight that was adjusted biweekly during the experiment period. Feed residues were collected 1 h after feeding. Wastes in the experimental tanks were siphoned 2 h prior to and after feeding. Approximately one-quarter to one-half of the tank water was replaced by fresh water daily.

**TABLE 1**
**Proportion (%) of Various Components in the Experimental Diets (Mean ± SD)**

| Ingredient (%) | Treatment | | |
|---|---|---|---|
| | $CP_1$ | $CP_2$ | $CP_3$ |
| Fish meal | 6 | 6 | 6 |
| Soybean meal | 13.27 | 20.38 | 27.49 |
| Rapeseed meal | 10 | 10 | 10 |
| Cottonseed meal | 12 | 12 | 12 |
| Wheat bran | 12 | 12 | 12 |
| Corn | 6 | 6 | 6 |
| Wheat middling | 16 | 16 | 16 |
| Fish oil:soy oil (1:1) | 8.62 | 8.48 | 8.35 |
| Dicalcium phosphate | 1.5 | 1.5 | 1.5 |
| Vitamin premix | 0.06 | 0.06 | 0.06 |
| Mineral premix | 0.3 | 0.3 | 0.3 |
| Antioxidant | 0.015 | 0.015 | 0.015 |
| α-Cellulose (CMC) | 14.23 | 7.26 | 0.28 |
| **Nutrition (Dry Weight, %)** | | | |
| Crude protein | 22.09 ± 0.38 | 25.26 ± 0.38 | 28.35 ± 0.25 |
| Crude lipid | 10.16 ± 0.09 | 10.19 ± 0.09 | 10.28 ± 0.10 |
| Ash | 4.98 ± 0.15 | 5.52 ± 0.09 | 5.98 ± 0.03 |
| Moisture | 5.17 ± 0.66 | 5.05 ± 0.69 | 4.95 ± 0.74 |
| Gross energy* (KJ/g) | 15.44 ± 0.02 | 16.58 ± 0.03 | 17.70 ± 0.04 |

* Determined using a model XRY-1B oxygen bomb calorimeter.

Water used in the experiment was tap water exposed to 24-h aeration before use. Tank water was aerated 24 h/day to ensure high dissolved oxygen content (≥5 mg/l). During the experiment period, water temperature averaged at 28.1 ± 0.9°C and pH averaged at 7.5 ± 0.2. The experiment was conducted under natural photoperiod. Water temperature and dissolved oxygen were recorded during each feeding period.

## Sample Collection and Analyses

After the 56-day experiment, fish were starved for 24 h prior to blood sample collection. Three to five fish were randomly taken from each tank and blood samples were withdrawn from the tail area. Blood serum was centrifuged at 2000 rpm/min for 5 min and stored at –20°C prior to chemical analyses. Blood sugar and lipids from each sample were determined twice within 24 h of sampling using standard methods[9].

## Data Analyses

All experimental data were analyzed using SPSS statistical software (version 13.0). Two-way ANOVA and Duncan's multiple comparison were used to assess significant differences among treatments. Results were considered significant at $p < 0.05$.

## RESULTS

### Effects of Dietary Protein Level

Glucose and HDL-C contents in juvenile tilapia increased as the level of dietary protein increased (Fig. 1A and B) and were 7 and 13%, respectively, in the treatment group fed with 28% dietary protein, higher than those of the treatment groups fed with 22 and 25% dietary protein (Table 2). However, HDL-C contents between treatment groups fed with 22 and 25% dietary protein showed no significant difference (Table 2).

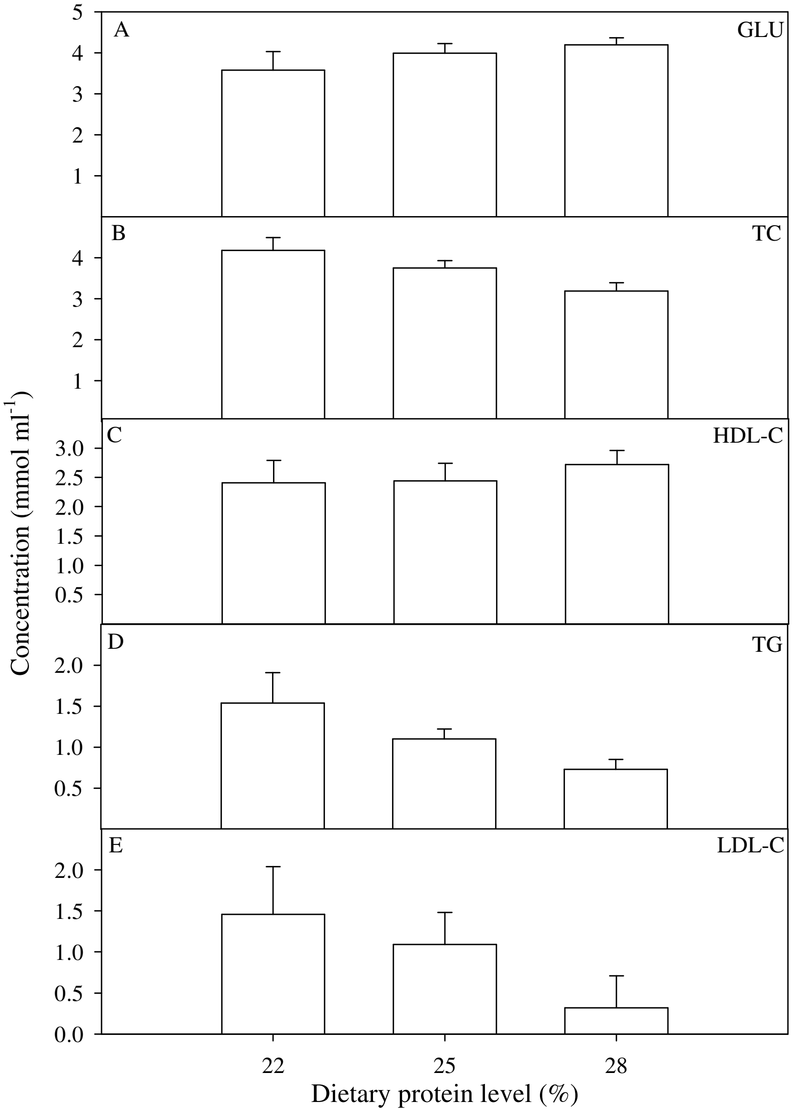

**FIGURE 1.** Effects of dietary protein levels on (A) GLU, (B) TC, (C) HDL-C, (D) TG, and (E) LDL-C of the new GIFT strain of juvenile Nile tilapia. Error bars represent one standard deviation of each treatment.

**TABLE 2**
**Results of ANOVA of the Effects of Dietary Protein and L-Carnitine on Blood Sugar and Lipid Contents of the New GIFT Strain of Juvenile Nile Tilapia**

| | Dietary Protein | | L-Carnitine | | Dietary Protein × L-Carnitine | |
|---|---|---|---|---|---|---|
| | F | *p* | F | *p* | F | *p* |
| GLU | 139.316 | 0.0001 | 66.044 | 0.0001 | 11.967 | 0.0001 |
| HDL-C | 13.544 | 0.0001 | 21.865 | 0.0001 | 2.553 | 0.056 |
| LDL-C | 94.832 | 0.0001 | 32.276 | 0.0001 | 2.059 | 0.109 |
| TC | 297.783 | 0.0001 | 31.943 | 0.0001 | 4.636 | 0.005 |
| TG | 233.422 | 0.0001 | 33.384 | 0.0001 | 7.359 | 0.001 |

Blood TC, TG, and LDL-C contents in juvenile tilapia followed a similar trend that decreased as dietary protein level increased (Fig. 1C–E). These parameters in the treatment group fed with 28% dietary protein were 24, 53, and 78% lower than the treatment group fed with 22% dietary protein (Table 2). Multiple comparison analysis indicated that there were significant differences in TC, TG, and LDL-C contents among the three treatment groups.

## Effects of L-Carnitine Level

The effects of L-carnitine on blood sugar and lipids were similar to those of dietary protein. GLU and HDL-C contents in juvenile tilapia were lowest in the treatment groups fed with diets without L-carnitine supplementation and highest in the treatment groups fed with diets with the highest L-carnitine supplementation (Fig. 2A and 2B), with the latter 20 and 36% greater than the former in GLU and HDL-C contents, respectively (Table 2). GLU and HDL-C contents in all treatment groups with L-carnitine supplementation were significantly higher than those of the control group (Table 2).

Contents of TC, TG, and LDL-C decreased as L-carnitine in diets decreased (Fig. 2C–E). Treatment groups with L-carnitine supplementation had lower TC, TG, and LDL-C contents than the control (Table 2). The lowest TC, TG, and LDL-C contents were found in the treatment groups with the highest L-carnitine (800 mg/kg) and were 12, 41, and 68% lower than the control.

## Interactive Effects of Dietary Protein and L-Carnitine Levels

Among the 15 treatment groups, juvenile tilapia in the treatment group $CP_{1-1}$ (22% protein and 0 mg/kg L-carnitine) had the lowest blood GLU and HDL-C contents (Fig. 3A and 3B), and the highest TC, TG, and LDL-C (Fig. 3C–E). Juvenile tilapia with the highest GLU and HDL-C were found in treatment groups $CP_{3-4}$ (28% protein and 600 mg/kg L-carnitine) and $CP_{1-5}$ (22% protein and 800 mg/kg L-carnitine), respectively. Compared to the treatment groups with the same level of dietary protein, L-carnitine supplementation significantly increased GLU and HDL-C by 11.45 and 47.52%, respectively ($p < 0.05$). The lowest levels of TC, TG, and LDL-C were found in treatment groups $CP_{3-2}$ (28% protein and 200 mg/kg L-carnitine), $CP_{3-5}$ (28% protein and 800 mg/kg L-carnitine), and $CP_{3-3}$ (28% protein and 400 mg/kg L-carnitine), respectively. Compared to the control group with the same level of dietary protein, TC, TG, and LDL-C levels were reduced by 13.84, 36.67, and 92.31% (Fig. 3). Two-way ANOVA indicates that there were significant effects of dietary protein and L-carnitine on GLU, TC, TG, HDL-C, and LDL-C in juvenile tilapia (Table 2). The cross-interactions of dietary protein and L-carnitine also had

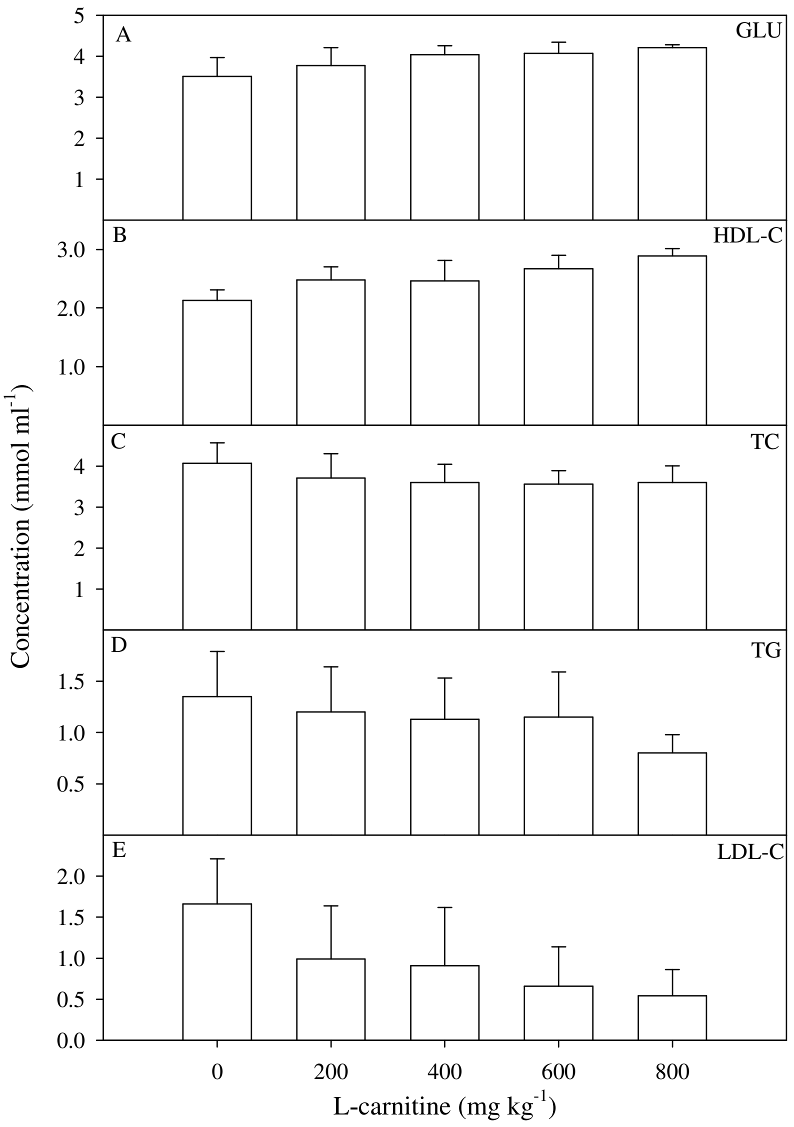

**FIGURE 2.** Effects of L-carnitine levels on (A) GLU, (B) HDL-C, (C) TC, (D) TG, and (E) LDL-C of the new GIFT strain of juvenile Nile tilapia. Error bars represent one standard deviation of each treatment.

significant effects on GLU, TC, and TG contents in the juvenile tilapia, but had no significant effects on HDL-C and LDL-C (Table 2).

## DISCUSSION

### Effects of Dietary Protein on Blood Sugar and Lipids in Tilapia

Blood sugar is primarily produced from glycogenolysis or gluconeogenesis in the liver. When a stomach is empty or under starvation, blood sugar in animals is produced by glycogenolysis and released to supply glucose for the central nerve system and red cells. However, glycogen will be nearly depleted after 24- to 48-h starvation. Under this circumstance, TG and cholesterol stored in the liver and blood are hydrolyzed by the fatty acid enzymes to form free-fatty acids and glycerol. These free-fatty acids and glycerol are transported into the liver and converted to coenzyme A (CoA), which is then transported into the

Research Article
*TheScientificWorldJOURNAL* (2009) 9, 1197–1205
ISSN 1537-744X; DOI 10.1100/tsw.2009.129

TheScientificWorldJOURNAL
www.thescientificworld.com

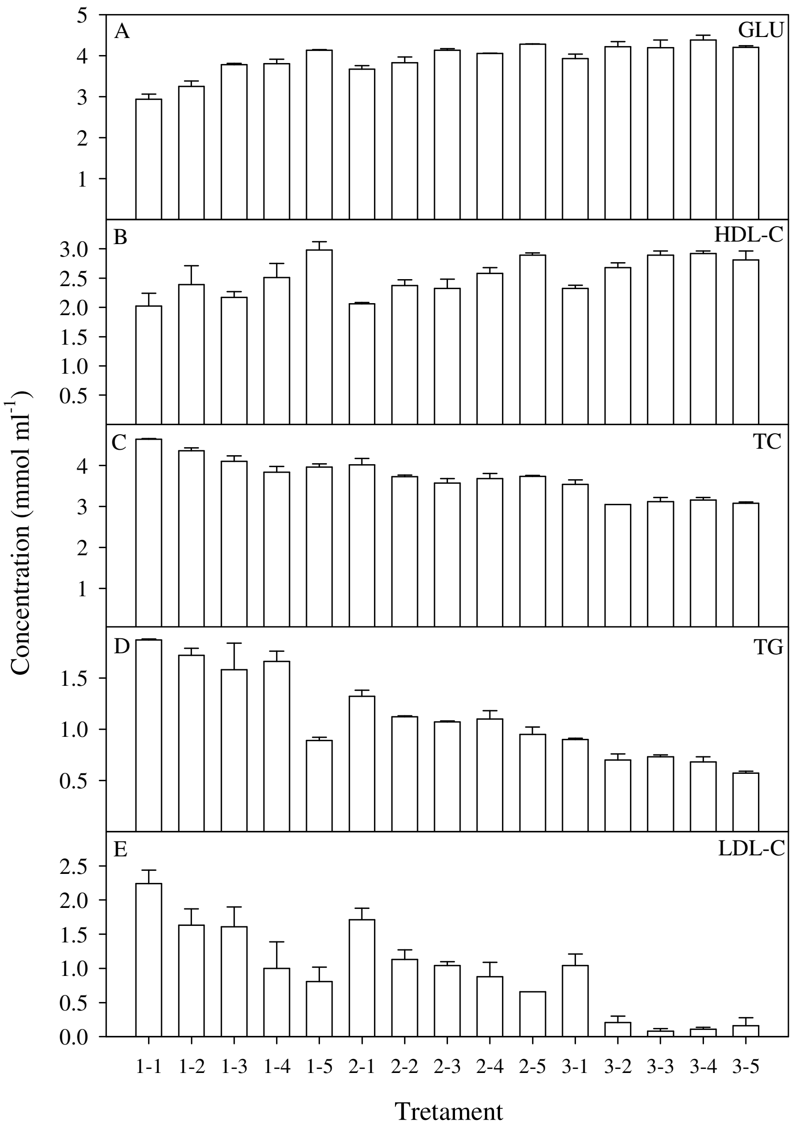

**FIGURE 3**. Interactive effects of dietary protein L-carnitine levels on (A) GLU, (B) HDL-C, (C) TC, (D) TG, and (E) LDL-C of the new GIFT strain of juvenile Nile tilapia. Error bars represent one standard deviation of each treatment. X-axis label represents the levels of protein and L-carnitine treatments.

mitochondria by acylcarnitine L-esters for β oxidation. Glycerol in the liver is converted to glucose via gluconeogenesis, which is then sent back to the blood and various tissues.

In the liver, LDL and cholesterol react to form LDL-C, which is transported to the blood. During starvation, the liver must oxidize TG and cholesterol to supply energy. As a result, the production of cholesterol and LDL-C are reduced. However, cholesterol requires HDL to form HDL-C that is subsequently transported into liver for oxidation and energy production. When starved, the liver will obtain TG and cholesterol from the blood and other tissues for energy production. Under this condition, the liver's ability for HDL synthesis is enhanced; the amount of HDL released to blood increases, and the HDL-C level in blood is also elevated[10]. The present experiment shows that blood sugar (GLU) and HDL-C contents in juvenile tilapia increased by 30 and 15%, respectively, as dietary protein level increased from 22 to 28%, while TC, TG, and LDL-C decreased by 20, 50, and 50%, respectively, as

*Corresponding author.
©2009 with author.
Published by TheScientificWorld; www.thescientificworld.com dietary protein level increased from 22 to 28% under the condition of no L-carnitine supplementation (Fig. 3). Similar results were reported previously on other species of fish[11].

Liver lipid content in fish fed with a high-protein diet was significantly lower than that in fish fed with a low-protein diet (Chen et al., unpublished data). When the stomach was empty, usable TG content in the liver was relatively low. Compared to fish fed with low dietary protein, fish fed with high dietary protein had higher requirements for blood TC and cholesterol. This leads to the marked decreases in blood TC and cholesterol contents in fish fed with high dietary protein. Furthermore, in comparison with fish fed with low dietary protein, the ability of cholesterol synthesis in fish fed with high dietary protein is relatively weak, but the ability of HDL synthesis is high. Therefore, in fish receiving a high-protein diet, the amount of LDL-C released to the blood is relatively low, but the content of HDL-C in blood increases. During starvation, fish fed with a high-protein diet require more TG from blood, which results in higher glycerol and subsequently higher GLU than fish fed with low-protein diets. This explains a positive relationship between dietary protein level and GLU and HDL-C level, and a negative relationship between dietary protein level and TG, TC, and LDL-C contents observed in the present experiment. Our results showed that TC and LDL-C contents decreased by 50% when dietary protein level increased from 22 to 28% with no L-carnitine supplementation (Fig. 3). Other studies also found a similar role of dietary protein on liver lipids. For example, Jauncey[12] found that the body fat level of juvenile tilapia (*Sarotherodon mossambicus*) decreased from 10 to 4% as dietary protein level increased from 0 to 56%. Yang et al.[11] showed that increasing dietary protein levels from 13 to 55% led to continuous reductions of liver glycogen and liver lipid contents in a juvenile cyprind fish (*Spinibarbus hollandi*).

## Effects of L-Carnitine on Blood Sugar and Lipids in Juvenile Tilapia

L-carnitine supplementation has been shown to reduce serum TC, TG, and LDL-C levels of obese rats[13,14], rabbits[5], laying hens[15], and fish[3,4,9,16] and also increased serum HDL-C level in obese rats[14]. These findings are in agreement with the present study, indicating that GLU and HDL-C increased and TC, TG, and LDL-C decreased as dietary L-carnitine increased in juvenile tilapia.

Our recent experiment shows that crude fat level in the liver and spleen of juvenile tilapia decreased as L-carnitine supplementation increased, and was significantly lower than those fed a diet without L-carnitine supplementation. This indicates that the controlling mechanisms of L-carnitine on blood sugar and lipids are similar to those of dietary protein. Ozório et al.[17] indicated that high-carnitine supplements increased total carnitine content and reduced tissue-free carnitine in African catfish (*Clarias gariepinus*). In a study of the effects of L-carnitine on fatty acid metabolism of avian broilers, Xu et al.[15] found that L-carnitine supplementation significantly increased the L-carnitine level in the liver and blood serum. L-carnitine is the sole carrier of long-chain fatty acids for entry into mitochondria. Increases in L-carnitine level in the liver and serum may accelerate the transport of acyl-CoA dehydrogenase to mitochondria[10,18] and, hence, enhance the oxidation of fatty acids and decrease liver and serum TG[1,2]. Previous studies have found that increases in dietary L-carnitine led to reductions of body fat content in various cultivated fish[3,4,6,16].

## Interactive Effects of Dietary Protein and L-Carnitine on Blood Sugar and Lipids in Juvenile Tilapia

Our data also showed that increasing both dietary protein and L-carnitine levels significantly affected blood sugar and lipid contents in the new GIFT strain of juvenile Nile tilapia. For example, at the dietary protein level of 22%, blood sugar (GLU) was 2.94 mmol/ml with no L-carnitine supplementation, but was 4.20 mmol/ml when L-carnitine supplementation was 800 mg/kg. However, the effect of L-carnitine decreased at higher dietary protein levels. For example, at the dietary protein level of 28%, GLU was 3.93 mmol/ml with 0 mg/kg of L-carnitine and 4.20 mmol/ml with 800 mg/kg of L-carnitine. Similar interactive effects were also observed for TC and TG. However, the interaction of dietary protein and L-

carnitine on HDL-C was not significant (Table 2). This suggests that increases in dietary protein or L-carnitine alone may lead to an effect on HDL-C similar to simultaneous increases of both variables. The least interactive effect of dietary protein and L-carnitine was found for LDL-C ($p$ = 0.109). Further studies are needed in order to assess the optimal levels of dietary protein and L-carnitine supplementation that results in the most significant changes in blood sugar and liver lipid contents in tilapia.

In summary, increases in the protein or L-carnitine level in diets can increase the levels of GLU and HDL-C and decrease TC, TG, LDL-C in the new GIFT strain of juvenile Nile tilapia. Simultaneous increases in dietary protein and L-carnitine levels resulted in further improvements of blood sugar and lipid contents of the new GIFT stain of juvenile Nile tilapia in the present experiment.
